# mPEG-PLGA Nanoparticles Labelled with Loaded or Conjugated Rhodamine-B for Potential Nose-to-Brain Delivery

**DOI:** 10.3390/pharmaceutics13091508

**Published:** 2021-09-18

**Authors:** Emanuela Fabiola Craparo, Teresa Musumeci, Angela Bonaccorso, Rosalia Pellitteri, Alessia Romeo, Irina Naletova, Lorena Maria Cucci, Gennara Cavallaro, Cristina Satriano

**Affiliations:** 1 Department of Biological, Chemical and Pharmaceutical Science and Technologies (STEBICEF), 90123 Palermo, Italy; emanuela.craparo@unipa.it (E.F.C.); gennara.cavallaro@unipa.it (G.C.); 2 Department of Drug and Health Sciences, University of Catania, 95125 Catania, Italy; abonaccorso@unict.it (A.B.); alessia.romeo@virgilio.it (A.R.); 3 Institute for Biomedical Research and Innovation, National Research Council, 95126 Catania, Italy; rosalia.pellitteri@cnr.it; 4 PhD in Neuroscience, Department of Biomedical and Biotechnological Sciences, School of Medicine, University of Catania, 95125 Catania, Italy; 5 Inter-University Consortium for Research on the Chemistry of Metal Ions in Biological Systems, University of Bari, 70126 Bari, Italy; irina.naletova@ic.cnr.it (I.N.); cristina.satriano@unict.it (C.S.); 6 Institute of Crystallography, Research National Council, 95126 Catania, Italy; 7 Department of Chemical Science, University of Catania, 95125 Catania, Italy; lorena.cucci@unict.it

**Keywords:** fluorescent dye, olfactory ensheathing cells, PC12 cell line, co-polymers, nanomedicine, imaging

## Abstract

Nowdays, neurodegenerative diseases represent a great challenge from both the therapeutic and diagnostic points of view. Indeed, several physiological barriers of the body, including the blood brain barrier (BBB), nasal, dermal, and intestinal barriers, interpose between the development of new drugs and their effective administration to reach the target organ or target cells at therapeutic concentrations. Currently, the nose-to-brain delivery with nanoformulations specifically designed for intranasal administration is a strategy widely investigated with the goal to reach the brain while bypassing the BBB. To produce nanosystems suitable to study both in vitro and/or in vivo cells trafficking for potential nose-to-brain delivery route, we prepared and characterized two types of fluorescent poly(ethylene glycol)-methyl-ether-block-poly(lactide-*co*-glycolide) (PLGA–PEG) nanoparticles (PNPs), i.e., Rhodamine B (RhB) dye loaded- and grafted- PNPs, respectively. The latter were produced by blending into the PLGA–PEG matrix a RhB-labeled polyaspartamide/polylactide graft copolymer to ensure a stable fluorescence during the time of analysis. Photon correlation spectroscopy (PCS), UV-visible (UV-vis) spectroscopies, differential scanning calorimetry (DSC), atomic force microscopy (AFM) were used to characterize the RhB-loaded and RhB-grafted PNPs. To assess their potential use for brain targeting, cytotoxicity tests were carried out on olfactory ensheathing cells (OECs) and neuron-like differentiated PC12 cells. Both PNP types showed mean sizes suitable for nose-to-brain delivery (<200 nm, PDI < 0.3) and were not cytotoxic toward OECs in the concentration range tested, while a reduction in the viability on PC12 cells was found when higher concentrations of nanomedicines were used. Both the RhB-labelled NPs are suitable drug carrier models for exploring cellular trafficking in nose-to-brain delivery for short-time or long-term studies.

## 1. Introduction

The intranasal (IN) drug administration route represents an intriguing strategy for obtaining the rapid delivery of the drugs to the central nervous systems (CNS), by allowing the drugs to reach the brain directly. Such a route of administration overcomes the well-known limits presented by blood brain barrier (BBB) [1,2]. First pioneering studies on the IN administration strategy for bypassing the BBB were carried out by William Frey II in 1989. Since then, with the aim of achieving direct access into the brain, innovative devices have been developed and marketed (Optinose^®^, Bi-Directional™ technology), to drive drugs to the olfactory region in the upper site of the nose. It is also well-known that several pathways can be involved for drugs administrated through IN route [3,4,5].

Despite there being several studies on the use of free drugs through nose-to-brain delivery, it has been demonstrated that most molecules do not have suitable properties to reach therapeutic doses in the brain. Such reduced bioavailability can be due to the low instilled volumes that can be given intranasally, and/or to the local physiological mechanisms that reduce the drug’s access to the target site, such as the mucociliary clearance (which does not allow long residence time in the nose), or enzymatic degradation, besides temporary local disfunction (allergies, influenzae) altering the physiological function.

According to these premises, nanomedicine may offer new solutions to overcome these drawbacks of traditional administration routes to the CNS. Indeed, micro- and nano-emulsions, lipidic and polymeric NPs are widely investigated drug-delivery systems to load drugs commonly used for neurological disorders [6]. Specifically, the most studied materials to obtain polymer-based NPs for nose-to-brain delivery are chitosan and its derivatives, poly-lactide (PLA), poly-lactide-*co*-glycolide (PLGA), and their PEGylated derivatives [7,8].

In vitro and in vivo investigations are fundamental to understanding the potential of NPs for nanomedical application [9]. The difficulty in having an easy and effective labeling method to track nanomedicine represents the bottleneck for the developments of these formulations. Generally, fluorescent molecules were used to define well the intracellular trafficking and biodistribution fates of nanocarriers after IN administration. Encapsulation of dye into nanosystems is a widely used strategy to label them, allowing the in vivo and in vitro fate of the systems. Several advantages are associated loading the dye into the NPs: the dye signal is retained for more time due to its protection into nanocarriers; the nanocarrier can be used to encapsulate other substances or to functionalize the surface with target moiety. Referring to literature different hypothesis were disseminated, and it is a challenge the selection of most suitable dye (in term of physic-chemical properties) related to nanosystems that should be correlated also to the specific technique. A fluorescent molecule is adopted to the specific analysis, the related question is: is it possible to use it to detect NPs? And what method is it appropriately? For example, the 1-1′-dioctadecyl-3,3,3′,3′-tetra-methylindotricarbocyanine iodide (DiR), a lipophilic dye, is suitable for in vivo biodistribution using Fluorescence Molecular Tomography system; it was loaded into PLGA NPs in a recent investigation [10,11]. Other dye extensively used is Rhodamine B (RhB) as fluorescent for in vitro and/or in vivo studies in nanomedicine field [12,13,14,15]. After the identification of a suitable dye, the researcher defines the type of fluorescent NPs that should be prepared: dye can be covalently attached to a polymer, which is then blended with other material to obtain nano-formulations, or it can be encapsulated into NPs as a free moiety. Both approaches are widely used, with respective advantage and disadvantages for tracking NPs.

In this work, we have developed nanomedicines suitable for nose-to-brain delivery, increasing the efficiency of the system in terms of bioavailability and capability of translocation in the brain, PLGA–PEG was chosen as raw material to prepare NPs. PLGA–PEG is a promising raw material to prepare nanomedicine [8] and it is classified as a mucus-penetrating polymer, owing to the presence of the PEG portion, in fact, mucoadhesive properties could be increased with this type of material due to its penetrating action [16]. RhB was chosen as fluorescent dye, and to produce fluorescent NPs, RhB was entrapped by following two different methods, as free or after conjugation to a polyaspartamide/polylactide graft copolymer [12]. We obtained, respectively, RhB-loaded PLGA–PEG NPs (thereafter named loaded-PNP) or amphiphilic copolymer bearing RhB moieties blended with PLGA–PEG (thereafter named grafted-PNP), which were properly purified and characterized. Firstly, we selected the suitable purification methods for loaded-PNPs through physicochemical and technological characterizations. Release-profile studies were carried out to prove the entrapment of RhB in PNPs for all experiments.

The two selected nanomedicine were compared in the respects of mean size, polydispersity index (PDI) and zeta potential (ZP) through photon correlation spectroscopy (PCS. Differential scanning calorimetry (DSC) for thermometric evaluation and atomic force microscopy were performed to evaluate mean size of studied NPs in terms of core structure. NMR was used to give information about the conformation of PEG on PNPs surface.

Cytotoxicity evaluation through MTT assays of both systems was performed on olfactory ensheathing cells (OECs) and neuronal PC12 cells to assess their potential use for nose-to-brain delivery.

## 2. Materials and Methods

### 2.1. Materials

Poly(ethylene glycol) methyl ether-block-poly(lactide-*co*-glycolide) (mPEG-*b*-PLGA, PEG average Mn 5000, PLGA Mn 55,000), polyoxyethylene sorbitan monooleate (Tween^®^ 80) and RhB were purchased from Sigma Aldrich (Milan, Italy). All other chemical reagents used solvents and deionized water were of analytical grade. Ultrapure water was used throughout this study.

### 2.2. Synthesis of PHEA–g–RhB–g–PLA (Fluo-P)

α,β-Poly(N-2-hydroxyethyl)-d,l-aspartamide (PHEA), PHEA-*g*-RhB and PHEA-*g*-RhB-*g*-PLA were properly synthesized by following procedures already reported in literature [12,17,18].

^1^H-NMR spectra were registered by a Bruker Avance II-300 spectrometer, working at 300 MHz (Bruker, Milan, Italy).

Both PHEA and PHEA–*g*–RhB ^1^H–NMR spectra in D_2_O were superimposed with those reported in previously published papers, and the derivatization degree with RhB (DD_RhB_), resulted about 0.55 ± 0.05 mol% [18].

PHEA-*g*-RhB-*g*-PLA ^1^H–NMR (300 MHz, [D7].DMF, 25 °C, TMS): δ 1.15 (m, 12H_RhB_ CH_3_CH_2_–); δ 1.3 and δ 1.7 (2d, 3H_PLA_ –[OCOCH(CH_3_)]_194_–); δ 2.8 (m, 2H_PHEA_ –COCHCH_2_CONH–); δ 3.3 (t, 2H_PHEA_ –NHCH_2_CH_2_O–); δ 3.59 (t, 2H_PHEA_ –NHCH_2_CH_2_O–); δ 4.2–4.5 and δ 5.1–5.5 (m, 1H_PLA_–[OCOCH(CH_3_)]_194_–), and δ 4.8 (m, 1H_PHEA_ –NHCH(CO)CH_2_–); δ 7.0–8.0 (m, 10H_RhB_ H–Ar). The degree of derivatization in PLA (DD_PLA_), determined from the ^1^H-NMR spectrum, as reported elsewhere, was equal to 4.1 ± 0.46 mol% [18].

The weight-average molecular weight (${\bar{M}}_{w}$) of PHEA, PHEA–g–RhB and PHEA–RhB–PLA graft copolymers used in this study, were calculated from SEC chromatograms, resulting respectively 53.6 kDa (${\bar{M}}_{w}/{\bar{M}}_{n}$ = 1.2), 52.5 Da (${\bar{M}}_{w}/{\bar{M}}_{n}$ = 1.6), and 209.0 kDa (${\bar{M}}_{w}/{\bar{M}}_{n}$ = 1.50).

### 2.3. Preparation of Fluorescent Nanoparticles

#### 2.3.1. Rhodamine-B Loaded PLGA–PEG Nanoparticles (Loaded-PNP)

RhB loaded PLGA–PEG NPs were prepared by the “nanoprecipitation method” with modification as previously reported by Bonaccorso et al., [19].

PLGA–PEG (12 mg/mL) was dissolved in the organic phase (acetone). The aqueous phase (water/ethanol 1:1 *v*/*v*) was composed of Tween 80^®^ (0.1 *w*/*v*). RhB was added to aqueous phase at 0.005% *w*/*v* prior precipitation process. The organic phase was added dropwise under constant stirring at room temperature into the aqueous phase (volume ratio 1:2) until a milky suspension had formed. The organic solvent was removed under vacuum (Büchi R 111), (38–40 °C and 450–500 bar).

#### 2.3.2. PLGA–PEG and PHEA–g–RhB–g–PLA Blended Nanoparticles (Grafted-PNP)

This sample was obtained by dissolving in the organic phase PLGA–PEG and PHEA-*g*-RhB-*g*-PLA graft copolymer 5% *w*/*w* on the PLGA–PEG weight, and by following the procedure described above for loaded-PNPs.

An unlabeled sample (without RhB and PHEA-*g*-RhB-*g*-PLA) was also prepared as control (PNP).

### 2.4. Purification Procedures to Remove Unloaded RhB from Loaded-PNP

A purification process was used for loaded-PNP. Dialysis and centrifugation were investigated to eliminate any residual of surfactant and/or unloaded fluorescent molecules. The NPs suspensions were studied in terms of mean size, PDI and surface charge, before and after the purification phases, to evaluate variations due to the purification processes.

For centrifugation process we used Thermo-scientific SL 16R Centrifuge (Thermo Scientific Inc., Waltham, MA, USA) at 12,000 rpm for 1 h at 8 °C. The obtained supernatants were collected for HPLC analysis, pellet was resuspended in water and characterized through PCS analysis.

For dialysis process we used membranes (Mwco 3000 Da, diameter 11.5 mm; Spectra/Por^®^) previously hydrated. The colloidal suspensions inserted in dialysis membranes were immersed in 500 mL of distilled water. The frequencies of water changes per hour in dialysis (sample PNPs and loaded-PNP) were 3/3 L/h (3 L in 3 h). At the end of the procedure the samples were collected and characterized through PCS analysis.

Further centrifugation process was carried out for collected dialyzed samples to evaluate the encapsulation efficiency (EE%) and RhB release profile. For this aim the dialyzed samples were centrifuged at 12,000 rpm for 1 h at 8 °C, the obtained supernatant and pellet were analysed by UV.

### 2.5. Entrapment Efficiency of RhB into Loaded-PNP

The amount of free RhB in the loaded-PNPs was calculated to determine the EE%. The pellet obtained by ultra-centrifugated sample was dissolved in acetone. The amount of RhB in the supernatant was determined spectrophotometrically using a spectrophotometer (UV–VIS 1601 spectrophotometer, Shimadzu Italia, Milan, Italy) at a wavelength of 547 nm. The calibration curve for the quantitative evaluation of RhB in acetone was linear in the following range: 12.70–0.72 µg/mL (R^2^ = 0.9663). The amount of RhB in the supernatant was determined. The calibration curve for the quantitative evaluation of RhB in H_2_O/EtOH was linear in the following range: from 8.16 to 0.16 µg/mL at RhB λ max 547 (R^2^ = 0.9663). The *EE%* was calculated using the following Equation (1):

(1) $$\%E.E.=\frac{w_{i}-w_{pellet}}{w_{i}}\times 100$$

where *W_i_* is the amount of RhB added during preparation and *W_pellet_* is the amount of RhB determined in the pellet after dissolution in acetone.

### 2.6. Yield of Purification Process

In order to determine the most efficient purification methods we investigated the purification yield for both studied procedures. Purification efficiency was expressed as the percentage amount of dialyzed RhB compared with the unencapsulated amount.

In the case of dialyzed samples, a further centrifugation step was required.

The concentration of RhB in the obtained supernatants was measured by UV Spectroscopy. The percentage of purification was calculated using the following Equation (2):

(2) $$Purification\;efficacy\left(\%\right)=\frac{\mu gRhB\;supernatant}{\mu gRhB\;tot-\mu gRhB\;encapsulated}\times 100$$

Each experiment was performed in triplicate and the results represent the mean ± standard deviation (SD).

### 2.7. Particle Size Distribution and Zeta Potential Measurements

Nanocarriers’ mean size, polydispersity index (PDI) and ZP were determined by PCS (Zetasizer Nano S90; Malvern Instruments, Malvern, UK). The experiments were carried out at a detection angle of 90°, at 25 °C with a 4 mW laser operating at 633 nm as light source.

Each value was measured in triplicate. The results are shown as the mean ± SD.

### 2.8. In Vitro Release Profile of RhB from Loaded-PNPs

The amount of RhB released from loaded-PNPs was measured on the resuspended pellet obtained after centrifugation of the samples, the supernatants were analysed through UV and 1 mL of phosphate-buffered saline (PBS) (pH 7.4) was used to resuspend the pellets. Cellulose membrane dialysis tubing (MWCO 3.5 kDa, Flat width 18 mm, diameter 11.5 mm; Spectra/Por^®^ Dialysis Membrane, Waltham, MA, USA) containing the nanosuspensions were incubated in 20 mL of PBS at pH 7.4) and were maintained under magnetic stirring at 37 °C, up to 72 h. Release medium (1 mL) was took out at different time points (0, 1, 5, 24, 48, 72) and replaced with the same volume of fresh medium, to maintain the sink condition. To determine the RhB concentration in the collected samples UV analysis was used, the wavelength was 553 nm. Each formulation was analysed in triplicate.

Calibration curves for the quantitative evaluation of the probe were linear in the following ranges: (i) 6.00–0.17 µg/mL of RhB (R^2^ = 0.997) for analyses in PBS pH 7.4. This data was also used for in vitro release experiments.

### 2.9. Atomic Force Microscopy (AFM)

AFM imaging was performed on adlayers prepared by drop casting at room temperature on freshly cleaved muscovite mica (Ted Pella, Inc., Redding, CA, USA). Briefly, a 10 µL volume of the concentrated dispersion of NPs was deposited on the mica substrates and, after 5 min, the mica surface was washed with 1 mL of milli-Q water, dried under a gentle nitrogen stream and imaged. A Cypher AFM instrument (Asylum Research, Oxford Instruments, Santa Barbara, CA, USA) equipped with a scanner at an XY scan range of 30/40 μm (closed/open loop) was operated, in AC-mode, in air. Tetrahedral tips made of silicon and mounted on rectangular cantilevers (30 μm) were purchased from Olympus (AT240TS, Oxford Instruments, Santa Barbara, CA, USA). Images, with the surfaces from 1 to 10 μm^2^, were scanned and the sizes of particles were measured using a free tool in the MFP-3DTM offline section analysis software.

### 2.10. Differential Scanning Calorimetry (DSC)

Differential scanning calorimetry studies were carried out with a Mettler Toledo DSC 1 STARe system equipped with a Poly-Science temperature controller (PolyScience, Columbus, OH, USA). The sensitivity was automatically chosen as the maximum possible through the calorimetric system, and the reference was an empty pan (signal time constant 18 s; digital resolution of the measurement signal < 0.04 µW, calorimetric resolution 0.12 and sensitivity 11 both determined through the TAWN test; the sampling rate 50 values/second). Calibration was carried out using indium as described in the procedure of the DSC 1 Mettler TA STARe instrument. Raw materials, RhB, PNPa, loaded- and grafted-PNPa were sealed in an aluminum pan and submitted to DSC analysis to determinate the thermotropic parameters of samples. Each sample was submitted to heating and cooling cycles in the temperature range 10–200 °C at a scanning rate of 5 °C/min (heating) and at a scanning rate of 10 °C/min (cooling). Transition temperature was calculated from peak areas with the Mettler STARe Evaluation software system (version 16.20).

### 2.11. Spectroscopic Quantification of RhB Dye Loading in the PNPs

To quantify the amount RhB immobilized by the two different approaches, the molar extinction coefficient (ε) of RhB in ultrapure MilliQ water was determined, as shown in Supplementary Figure S1. A calibration curve was obtained by using five different dilutions, in the concentration range from 3.11 × 10^−6^ M to 5.18 × 10^−6^ M, of a stock 2.08 × 10^−3^ M solution prepared by dissolving 1 × 10^−3^ g of RhB in 1 mL of Milli-Q water. The linear regression of the maximum absorbance values recorded at the wavelength of 552 nm (R^2^ = 0.99823) resulted in the ε value of 4.5 × 10^4^ M^−1^cm^−1^.

The NPs purified either by dialysis or ultracentrifugation, were characterized by UV-vis spectroscopy on a Perkin Elmer UV-vis spectrometer (Lambda 2S) using quartz cuvettes with an optical path length of 1 cm, for the suspensions, and 0.1 cm, for the pellets. The spectra were recorded by diluting the suspensions, in MilliQ water, 45 and 3 times, respectively for the loaded-PNP and the grafted-PNP. Whereas the pellets derived by centrifugation or samples obtained by dialysis were diluted 100 and 8 times, respectively for the loaded-PNP and the grafted-PNP, respectively.

### 2.12. Determination of Surface PEG Density and PEG Chains Conformation on Nanoparticle Surface

The amount of PEG exposed on the nanoparticle surface, expressed as surface PEG density, was determined on the freeze-dried samples by following an already reported ^1^H NMR method [20,21].

First, PEG dispersions in D_2_O (range of concentrations: 10^−5^–10^−3^ M), containing ethylendiamine (EDA) (1 µL/mL) as internal standard, were analysed to set up a calibration by using the PEG integral values at δ 3.6 ppm (y = 8314.9x, R_2_ = 0.9993). Each sample of NPs was dispersed in D_2_O (12.5 mg/mL), the internal standard was added, and from the integral value of PEG found in the acquired spectrum, the amount of PEG was calculated by using the calibration curve.

By proper calculations and considering that the PEG chains exposed on the surface were full length of 5 kDa PEG, the number of PEG molecules per 100 nm^2^ of NPs surface area was determined by using the following Equation (3) and expressed as the surface PEG density [Γ] parameter:

(3) $$\left[\Gamma \right]=\left[\frac{M_{\mathrm{PEG}}\times 6.02\times 10^{23}}{\frac{W_{NP}}{d_{NP}}/\frac{4}{3}\pi {\left(D/2\right)}^{3}}/4\pi {\left(D/2\right)}^{2}\right]\times 100$$

where *M*_PEG_ is the total *PEG* content, *W_NP_* is the total mass of *NP*s, *d_NP_* is the density of nanoparticle (here we assume the density of *NP*s is equal to the density of polymer, 1.34 g/mL for PLGA 50:50), D is the particle diameter as measured by the dynamic light scattering.

Then, the surface area occupied by a single PEG chain could be calculated, considering that a single PEG chain occupies an area at the interface given by a sphere of diameter ξ, as elsewhere reported [21], following Equation (4):

(4) $$\xi \;\left(\mathrm{nm}\right)=0.076\times \sqrt{m_{\mathrm{PEG}}}$$

where m_PEG_ is the PEG Mw.

For PEG chains of 5 kDa ξ was found to be 5.4 nm and occupies an area that is 22.7 nm^2^. Therefore, considering a NPs surface area of 100 nm^2^, [Γ*], the number of unconstrained PEG molecules need to cover it was found to be 4.4.

### 2.13. Cellular Experiments

#### 2.13.1. Cell Culture Maintenance

RPMI1640 medium, streptomycin, L-glutamine, fetal bovine serum (FBS) and horse serum (HS) were provided by Sigma-Aldrich (St. Louis, MO, USA). NGF was obtained from Invitrogen Laboratories (Paisley, UK). Rat pheochromocytoma (PC12 line) cells were cultivated in complete medium, i.e., RPMI1640 supplemented with 10% HS, 5% FBS, 2 mM l-glutamine, 50 U/mL penicillin, and 50 µg/mL streptomycin. The cell culture was grown in tissue-culture treated Corning^®^ flasks (Sigma-Aldrich, St. Louis, MO, USA) in humidified atmosphere (5% CO_2_) at 37 °C (Heraeus Hera Cell 150C incubator).

#### 2.13.2. Differentiation of PC12 Cells

Differentiation of PC12 cells were performed, as described in the literature [22], with some modifications. PC12 cells were plated onto 48-well tissue culture plates coated with 0.01% poly-lysine according to the manufacturer-recommended procedure. Cells were plated at a density 3 × 10^4^ cells/well in complete medium and after 20 h of plating, the medium was replaced with RPMI1640 medium supplemented with 50 ng/mL NGF, 0.5% of HS, 0.25% of FBS, 2mM L-glutamine, 50 U/mL penicillin, and 50 µg/mL streptomycin. At day 2, 4, and 6 a concentrated stock of NGF was added for a final concentration of 50 ng/mL.

#### 2.13.3. Cytotoxicity Assays

3-(4,5-dimethylthiazol-2-yl)-2,5-diphenyltetrazolium bromide was purchased from Sigma-Aldrich (St. Louis, MO, USA). The effect of NPs on viability of differentiated PC12 cells was tested by incubation with the compounds, namely PNP, loaded-PNPs at the RhB concentrations of 2.1 × 10^−3^ and grafted-PNP at the RhB concentration of 1.1 × 10^−3^ M (concentrations for each), by diluting the samples with final polymer concentrations equal to 1.5 mg/mL, 3 mg/mL, 15 mg/mL from the purified pellets. The viable cells were quantified by the reaction with 3-(4,5-dimethylthiazol-2-yl)-2,5-diphenyltetrazolium bromide. After 90 min, the reaction was stopped by adding DMSO, and absorbance was measured at 570 nm (Varioskan^®^ Flash Spectral Scanning Multimode Readers, Thermo Scientific, Waltham, MA, USA). Results were expressed as percentage of viable cells over the concentration of each compound. The experiments were repeated in triplicate and results expressed as mean ± SEM.

#### 2.13.4. ROS Assays

To analyze the intracellular ROS production, differentiated PC12 cells were treated with NPs for 24 h and then stained for 15 min with 2′,7′-Dichlorofluorescein (DCF) and Hoechst33342. Fluorescence of the samples was analyzed by fluorescent plate reader (Varioskan^®^ Flash Spectral Scanning Multimode Readers, Thermo Scientific, Waltham, MA, USA). Results are represented as the increase in DCF, normalized with live-cell fluorescent staining of DNA Hoechst33342, with respect to controls and are means ± SEM for three wells for each treatment.

#### 2.13.5. Laser Scanning Confocal Microscopy Imaging

An Olympus FV1000 confocal laser scanning microscope (LSM), equipped with diode laser (405 nm, 50 mW) and gas lasers (multiline Argon: 457 nm, 488 nm, 515 nm, total 30 mW; HeNe(G): 543 nm, 1 mW and HeNe(R): 633 nm, 1 mW) was utilized to execute confocal microscopy studies. Images were collected with oil immersion objective (60xO PLAPO), in sequential mode, randomly all through the area of the well. The detector gain was fixed at a constant value. The image analysis was carried out by means of Huygens Essential software (by Scientific Volume Imaging B.V., Hilversum, The Netherlands).

To perform the experiments of cellular uptake, PC12 cells were plated in poly-lysine precoated glass bottom dishes (WillCo-dish^®^, Willco Wells, B.V., Amsterdam, The Netherlands) with 12 mm of glass diameter at a density of 1 × 10^4^ cells/well in complete medium, then differentiated according to the protocol described above. Thereafter, cells were treated for 2 h, at 37 °C into the incubator, under humidified atmosphere of air/CO_2_ (95:5) with RhB (4 µM) or PNPs (3 mg/mL), either bare or RhB-functionalized nanoparticles.

Afterwards, cells were stained with nuclear dye Hoechst33342 (1 μg/mL) and fixed with high purity 2% paraformaldehyde in PBS, pH = 7.3.

### 2.14. In Vitro Tests on OEC

#### 2.14.1. Animals

Experiments were carried out on 2-day-old mouse pups (P2), provided by Envigo RMS s.r.l. (Udine, Italy). Animals were kept in a controlled environment (23 ± 1 °C, 50 ± 5% humidity) with a 12 h light⁄dark cycle with food and water available ad libitum. Experiments were carried out in compliance with the Italian law on animal care no. 116⁄1992 and in accordance with the European Community Council Directive (86⁄609⁄EEC) and were approved by the Ethical Committee at the University of Catania (Italy). Efforts were made to minimize the number of animals used.

#### 2.14.2. Cell Cultures

OECs were isolated from pup’s olfactory bulbs as previous described by Pellitteri et al., [23]. Briefly, bulbs were removed from decapitated pups and dissected out in cold (+4 °C) Leibowitz L-15 medium. Then, the pellets were digested in Medium Essential Medium-H containing collagenase and trypsin. Trypsinization was stopped with Dulbecco’s Modified Eagle’s medium (DMEM) supplemented with 10% Fetal Bovine Serum (FBS). Cells were resuspended and plated in flasks, fed with fresh complete medium DMEM/FBS, 2 mM L-glutamine and antibiotics. To reduce the number of dividing fibroblasts, cytosine arabinoside (10^−5^ M), an antimitotic agent, was added 24 h after initial plating. Subsequently, an additional step transferring from one flask to a new one, to reduce contaminating cells, was carried out. Purified OECs were plated both on 25 cm^2^ flasks and cultured in DMEM/FBS at 37 °C.

#### 2.14.3. MTT Bioassay

When cells were confluent, they were detached by trypsin and re-plated in multiwell 24-well plates. After 24 h the NPs (PNP, loaded-PNP, grafted-PNP) were added in OEC cultures at different concentrations (0.1, 0.5, 1.0 e 5.0 mg/mL) and incubated at 37 °C in DMEM/FBS medium 24 h, for cell viability test. Some OECs were grown without the presence of PNPs, and they were considered as controls. The OEC morphology in all conditions was monitored and the images were captured by phase-contrast microscopy (Zeiss, Oberkochen, Germany) using a 20× lens.

After 24 h that the NPs placed inside OEC cultures, cellular viability was evaluated by the 3-[4,5-dimethylthiazol-2-yl)-2,5-diphenyl] tetrazolium bromide (MTT, Sigma, Milan, Italy) reduction assay, a quantitative colorimetric method was utilized to evaluate cellular cytotoxicity: MTT was added to each multiwell and placed for 2 h in a CO_2_ incubator. We removed DMEM/FBS and added MTT solvent (acid-isopropanol/SDS), the cells were shaked for 15 min. A multisKan reader at 570 nm was used to measure the absorbance [23]. The collected data were expressed as the percentage MTT reduction in comparison with control cells. Differences between OECs grown in presence or not of NPs were assessed using one-way analysis of variance (one-way ANOVA) followed by post hoc Holm–Sidak test.

### 2.15. Statistical Analysis

Statistical analysis was performed using Prism 6 (GraphPad Software 6.01, Inc., La Jolla, CA, USA).

For the statistical analysis, we used a one-way analysis of variance (ANOVA) followed by Tukey’s multiple comparisons test for the analysis of NPs mean size and PDI. Significance was defined as *p* < 0.05.

## 3. Results and Discussion

In this paper, we have produced RhB labelled PEG–PLGA NPs as potential nanostructured carriers to study the nose-to-brain pathway as an administration route for drugs. In particular, the nanoprecipitation method was followed to obtain NPs; two different strategies to load the fluorescent dye have been exploited, i.e., the physical entrapment of the free RhB into the PEG–PLGA matrix, or the blending of PEG–PLGA with a copolymer carrying RhB covalently linked on the polymeric backbone [12,18]. Being different entrapment strategies, two different nanocarriers were obtained, respectively named loaded-PNPs when the free dye was entrapped and grafted-PNPs when the dye was entrapped into the nanoparticle matrix after conjugation with a graft copolymer.

### 3.1. Preparation and Evaluation of Physical-Chemical Properties of the Loaded-PNPs

In the case of loaded-PNPs, being the dye physically entrapped into PEG–PLGA matrix, a relevant question to solve was the determination of RhB release profile from the nanosystems in physiological conditions to prove that the fluorescence determined during the in vitro and in vivo experiments was due to the cargo and not to the dye released during the experiment. Thus, first step was to purify the nanoformulations to remove the excess of the surfactant and the unloaded RhB; for this purpose, we investigated two purification processes: dialysis and centrifugation. Physicochemical characterization and purification yield were performed through PCS and UV analysis to select the most suitable method (Table 1). Nanoprecipitation allowed to obtain NPs based on PLGA–PEG with a core constituted by the PLGA portion, the while PEG portion forms a hydrophilic corona, resulting in a core/corona structure. The PEG portion forms a flexible layer on the surface of the NPs, this thick layer is referred generally to as the “mushroom or brush pattern” as reported widely in literature [8,24]. Nanoprecipitation is usually selected due to its straightforwardness and user-friendliness with standard laboratory equipment. With this method, it is possible to obtain samples in small volumes, reducing the amount of starting raw materials.

**Figure 1:**
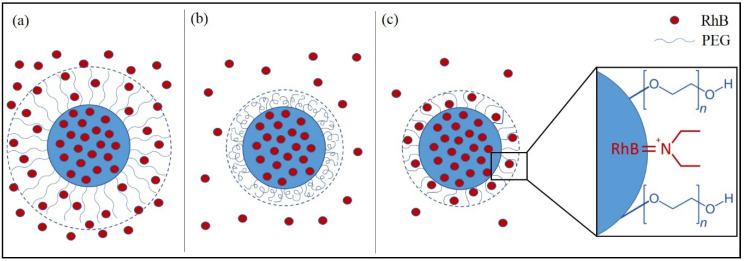
Scheme of possible conformation of loaded-PNPs before (**a**) and after purification processes with centrifugation (**b**) and dialysis (**c**).

The decrease of solubility of the polymer and/or the molecule that should be loaded in the solvent/water mixture is the first step to obtain particles, resulting in the formation of small aggregates at critical concentrations of super-saturation, allowing the realization of a homogeneous system. The growth of the aggregates occurs until they reach the state of colloidal stability. The probability of obtaining monodisperse particles depends on different factors; one is the uniform growth of the nuclei. It is important to strictly control these critical processes toavoid independent precipitation of the polymer and the molecule. In fact, this phenomenon should occur, we could obtain poor molecule loading and a dispersion that contains multiple species, such as polymeric particles without molecule, molecule-loaded polymeric particles, and molecule crystals. In this study, the nanosystem obtained before the purification process showed a mean size ~350.00 nm, representing the hydrodynamic diameter due to the extension of PEG chains as brush conformation (schematic representation in Figure 1a). Centrifugation decreased this parameter, due to the reduction of the hydrodynamic diameter represented by mushroom conformation (Figure 1b), owing to the subjected force. This phenomenon was also showed in dialysis process and in this case, it was due to the residual amount of RhB on the surface that influenced the conformation of PEG chains, as illustrated in the scheme of Figure 1c, the hypothesis was the formation of “pockets” that hold RhB on the surface.

The two purification processes allowed us to obtain monodisperse nanosystems, with an increase in the absolute values of ZP (from −1.4 to −23.4 mV). The variation of ZP values was related to surfactant remotion, and the difference for the nanosystems obtained by the two different purification processes was due to a residual amount of RhB that was not completely removed, in the case of dialysis, as confirmed by the yield-purification data (Table 1).

**Figure 2:**
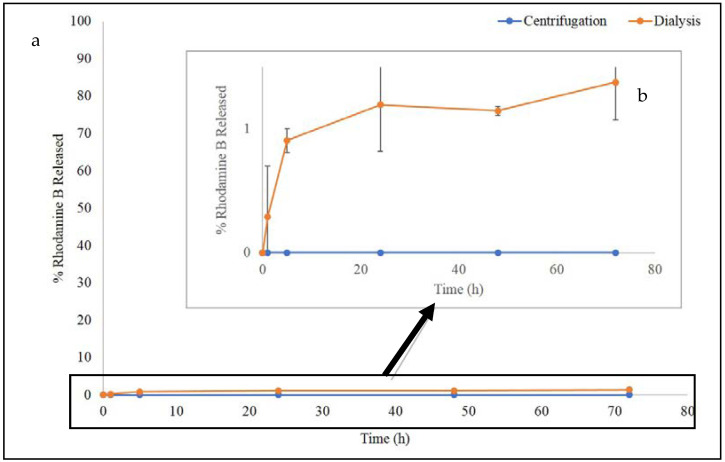
(**a**) Release profile of RhB from loaded-PNPs obtained from two different purification process, at 37 °C for 72 h; (**b**) Magnification of the 1% release profile.

In-vitro release studies (Figure 2) confirmed our previous hypothesis, in fact centrifugated samples did not show RhB release until 72 h, while dialyzed samples showed 1% of RhB released during the experiment. The low percentage (1%) of RhB released was probably due to the diffusion of RhB from the “pockets” and could be probably attributed to a conformational change of PEG tails. As previously demonstrated by Suna et al., the salt concentration of the release medium may weaken the hydrogen binding between PEG chains and water molecules. Consequently, PEG tails could stretch causing the collapse of “pockets” structures, resulting in the release of the dye that was previously retained [24,25].

Therefore, the centrifugation seems to be the most suitable purification process for loaded-PNPs to obtain NPs with RhB entrapped in the PLGA core (almost the 60 wt% of the theoretical), which limited the diffusion processes to 72 h.

### 3.2. Preparation of Grafted-PNP

**Figure 3:**
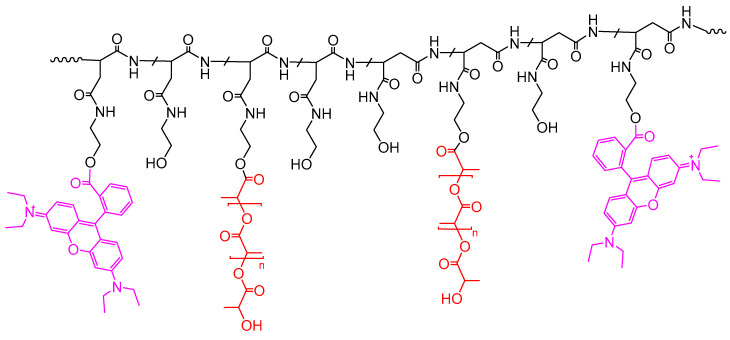
The chemical structure of PHEA–g–RhB–g–PLA graft copolymer (Fluo-P) (*n* = 194).

To obtain fluorescent PLGA–PEG NPs with the probe stably incorporated inside them a graft copolymer containing covalently linked RhB was blended into the NPs matrix. In particular, the labelled graft copolymer was obtained starting from α,β-Poly(*N*-2-hydroxyethyl)-d,l-aspartamide (PHEA) derivative, by functionalization with proper amount of RhB and polylactic acid (PLA), obtained the fluorescent PHEA–*g*–RhB–*g*–PLA graft copolymer [12,18,20]. Both these features had a very specific function: RhB made the polymer fluorescent, while PLA made it hydrophobic, ensuring the formation of insoluble nanostructures in aqueous media. It was already reported in the literature the great potential of the PHEA–*g*–RhB–*g*–PLA graft copolymer itself as starting material to produce several drug delivery systems (micro- and nano-structures) for active targeting and theranostic applications [12,18,20]. The chemical structure of PHEA–*g*–RhB–*g*–PLA graft copolymer was depicted in Figure 3. Chemical and enzymatic stability of fluorescent dye covalently linked to the copolymer backbone by ester linkage was demonstrated until 4 days of incubation [20].

### 3.3. Quantitative Evaluation of RhB Dye in the Loaded-PNPs and Grafted-PNP

To determine the amount of RhB entrapped into either loaded- or grafted-PNP after purification, and to confirm that centrifugation was the process more appropriate to recover both samples, both PEG–PLGA NPs labelled with RhB by physical incorporation (loaded-PNP) or by covalent conjugation (grafted-PNP), were characterized by UV-visible spectroscopy, by using the unlabelled PEG–PLGA nanoparticles (PNP) as control sample, using the two types of purification methods.

**Figure 4:**
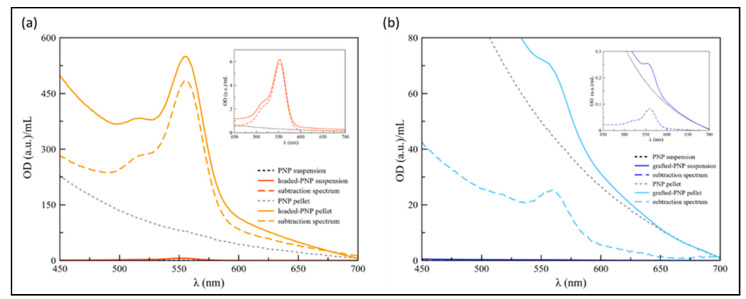
UV-vis optical density (OD) spectra of: (**a**) PNP (dotted black line) and loaded-PNPs (solid red line) suspensions, obtained after the dialysis process, compared with PNP (dotted grey line) and loaded-PNPs pellets obtained after the centrifugation steps; (**b**) PNP (dotted black line) and grafted-PNP (solid light blue line) suspensions, compared to PNP (dotted grey line) and grafted-PNP pellets. In the insets the magnified region for the suspensions. Dashed lines refer to the spectra obtained by the subtraction of the dyelabeled PNP spectra with that of unlabeled PNPs. Spectra were recorded by diluting the suspensions, in MilliQ water, 45 and 3 times, respectively for loaded-PNPs and grafted-PNP. Whereas, the pellets were diluted 100 and 8 times, for the loaded-PNPs and grafted-PNPs, respectively.

The spectra of loaded-PNPs and grafted-PNPs are displayed in Figure 4.

The difference spectra (dashed lines) were shown to remove the background effect from the PEG–PLGA nanosystems, including scattering from the NPs dispersion, as particularly evident for grafted-PNP samples (Figure 4). As to the PNP suspensions obtained after the dialysis process, the RhB characteristic absorbance peak was found at 552 and 560 nm for dialyzed loaded-PNPs (Figure 4A) and for the grafted-PNP (Figure 4B), respectively. Such a red shift (Δλ = 8 nm) confirmed the actual covalent interaction between PNP and dye for the grafted-PNP, with a limitation of freedom of rotational movement of the RhB molecules at the interface [26]. Based on the maximum of absorbance, the estimated RhB concentration was 2.6 × 10^−4^ M for the loaded-PNPs and 3.7 × 10^−6^ M for the grafted-PNP.

As to the PNP dispersions purified by centrifugation, the loaded-PNPs showed a red-shift of the RhB absorbance peak of about 3 nm (λmax = 555 nm) (Figure 4B), most likely due to the reorientation of the “disordered” layer of dye molecule physiosorbed on the particle surface, as an effect of the concentration procedure [27]. As to the grafted-PNPs, the particle dispersion continued to maintain the absorbance maximum of the dye at 560 nm, as expected for a stable chemisorbed layer of molecules surrounding the nanoparticle surface. The calculated concentration of RhB, based on the maximum of absorbance values were 2.1 × 10^−3^ M and 1.1 × 10^−4^ M, respectively for loaded-PNPs and grafted-PNP pellets.

Therefore, based on our results, centrifugation seems to be the more suitable process for the purification of both loaded- and grafted-PNPs, and for this reason particles used for further characterization were obtained by centrifugation method.

### 3.4. Loaded-PNPs and Grafted-PNPs: Physical Evaluations

**Figure 5:**
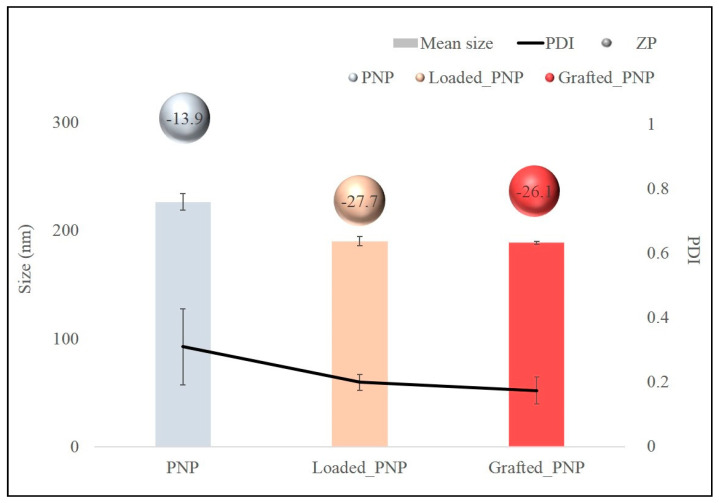
Comparative values in term of mean size (nm), PDI and ZP values (mV) of bare PNP; loaded-PNPs and grafted-PNPs.

In Figure 5, the physicochemical properties of the unloaded, loaded- and grafted-PNPs are depicted. The grafted-PNPs did not showed significant differences in terms of mean size (<200.0 nm) and PDI (<0.2) from the loaded-PNPs, while the unloaded-PNPs showed a significantly higher mean size. Both labelled PNPs also presented negative ZP values, with an increase in absolute value respective to the given PNP. The small decrease in terms of mean size, in the case of loaded-PNPs and grafted-PNPs with respect to unloaded ones, is probably due to the presence of RhB that helped to form a different matrix organization.

In Supplementary Figure S2 reported thermograms of raw materials and investigated PNP. Loaded-PNPs (yellow curve) and grafted-PNPs (brown curve) showed an endothermic peak at ~50 °C due to the typical endothermic peak of PCL polymer that occurred at about 57 °C.

**Figure 6:**
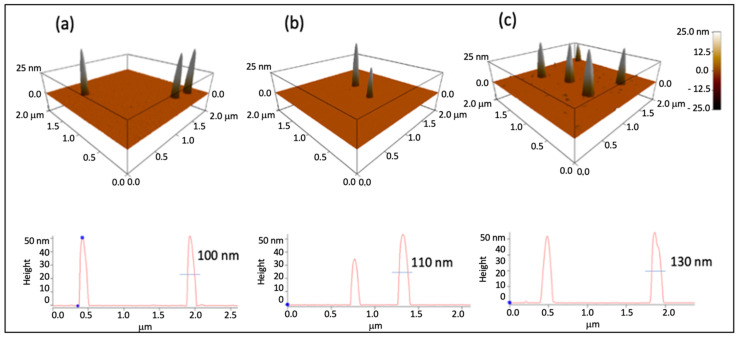
AFM 3D topography images, recorded in AC mode in air, for a PNP (**a**), a loaded-PNP (**b**) and a grafted-PNP (**c**) with the corresponding line-section curves.

AFM analyses displayed a general size increase for the labelled NPs, compared with the other PNPs, especially with grafted-PNPs (Figure 6). Such a data further proved the effective functionalization via both physisorption and chemisorption of PNPs with RhB, even if a reduced size of particles was shown respect to that estimated by dynamic light scattering (Figure 5). This fact was related to a collapse of the layer surrounding the nanoparticle surface, most likely due to the de-wetting process required for the AFM sample preparation. DLS, instead, measured the hydrodynamic size of the particle, which depends on both the particle “core” and the dye and solvation shell on the nanoparticle surface.

### 3.5. Surface PEG Density

The physicochemical properties, such as mean size, are fundamental aspects of ensuring the nose-to-brain drug delivery of colloidal systems. However, other surface properties could improve this process, such as the PEGylation degree. To evaluate the effective amount of PEG moieties on the surface of nanoparticle samples, a ^1^H NMR study was carried out [20]. A calibration curve was carried out with PEG solutions in D_2_O, in concentrations ranging between 10^−5^ and 10^−3^ M, by measuring the signal at 3.6 ppm and by using ethylenediamine (EDA) as internal standard.

By comparing the integrals of the PEG peaks in the spectra of nanoparticle sample dispersions in D_2_O to the calibration curve, the quantity of PEG on the NPs surface was found. The data, reported in Table 2, showed that no significant differences in the nanoparticle surface PEGylation were found to depend on the chemical composition and RhB presence and/or entrapment method.

Surface PEG density [Γ] (number of PEG per 100 nm^2^), calculated as described in the experimental part and reported in the Table 2, demonstrated a high surface PEG density of all obtained samples.

To assess the surface PEG density and PEG chains’ conformation on the NP surface, the number of unconstrained PEG molecules that occupy 100 nm^2^ of particle surface area ξ, was calculated and expressed as [Γ*]. Considering the Mw of PEG, it resulted to be 4.4 [21] [Γ/Γ*] is an index to measure the PEG density on the nanoparticle surface: values < 1 indicates low PEG density where PEG molecules are in a mushroom-like conformation; whereas values >1 indicate high PEG density where PEG molecules are in a brush-like conformation. For all samples, [Γ/Γ*] resulted to be higher than 1, indicating a high surface PEG density in all NPs, where PEG molecules were in a brush-like conformation. The PEGylation surface density of the NPs obtained by incorporating the P-Fluo graft copolymer seems to be quite similar to that of the systems obtained without RhB, while the use of free RhB seems to reduce PEG exposure (more leaning towards mushroom hypothesis due to the purification process), although, in all cases, this PEGylation was high enough to allow only the brush like conformation on the surface, with long, thin bristles of PEG extending from the NP surface when the sample was hydrated.

### 3.6. Biological Studies

#### 3.6.1. Cytotoxicity Study on OECs

**Figure 7:**
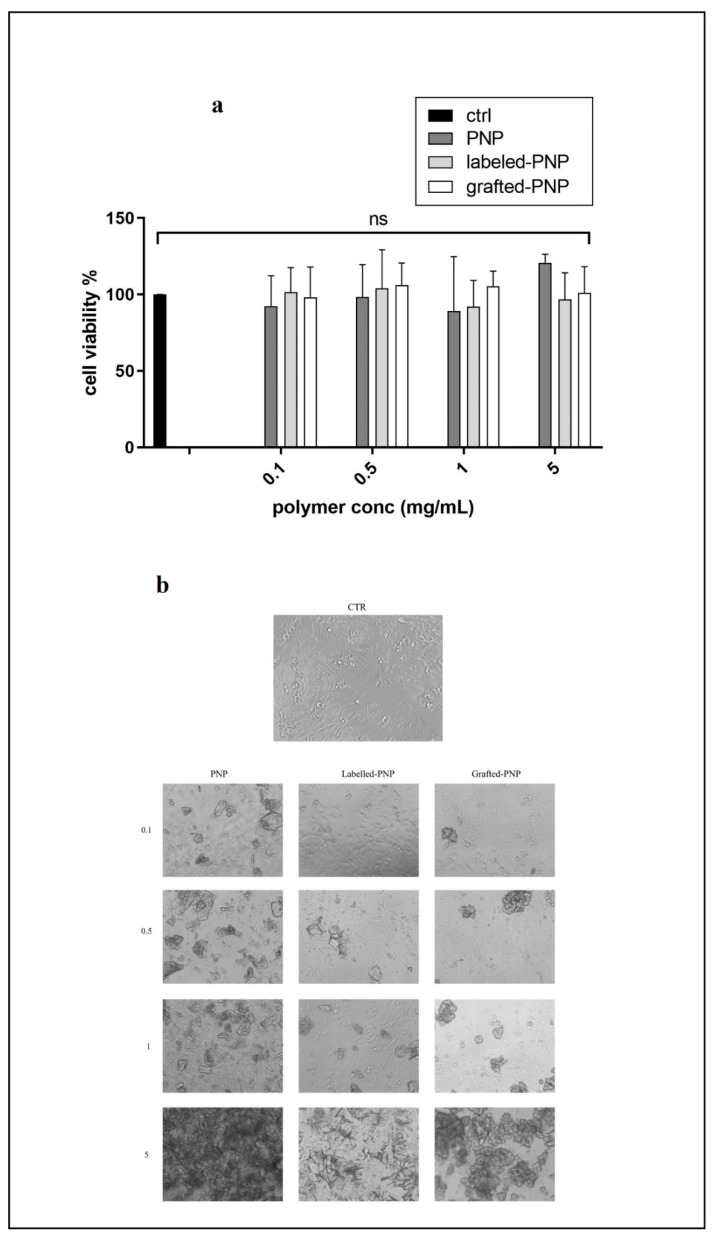
(**a**) Cell viability after exposure for 24 h of normal olfactory ensheathing cells (OECs) to PNPs, loded-PNPs and grafted-PNPs at different concentrations, ns means not significant statistically (**b**) Qualitative analysis by phase-contrast microscopy of representative fields of OECs, both as control and loaded to PNP, loaded-PNP and grafted-PNP at different concentrations (0.1, 0.5, 0.1 and 0.5 mg/mL).

The image 7A shows the OEC density grown with different NPs (PNP, loaded-PNP and grafted-PNP) at different concentrations (0.1, 0.5, 0.1 and 0.5 mg/mL). In Figure 7A are depicted cell viability percentages of the different nanosystems and in Figure 7B we showed the qualitative analysis by phase-contrast microscopy of representative fields of OECs. Our results demonstrated that PNPs were not cytotoxic toward OEC, as evidenced by no variation in cells’ viability, the differences between the type of PNPs were not significant for all concentrations tested (Figure 7). In this experiment, OECs were chosen because they represent a special glial population of the olfactory system that accompanies the unmyelinated olfactory axons of receptor neurons. Our previous investigations focused on the possible uptake of the different NPs into OECs to realize a drug carrier for intranasal administration used for nose-to-brain delivery. NPs could be switched with neuronal cells to reach the brain via an anterograde axonal pathway; this hypothesis should be deeply investigated [9].

#### 3.6.2. Cytotoxicity Effect on a Differentiated Pheochromocytoma Cell Line (d-PC12)

Dose-response effect on cell-growth and -death levels were performed to compare the cytotoxic activity of labelled NPs on a differentiated rat pheochromocytoma cell line (d-PC12).

**Figure 8:**
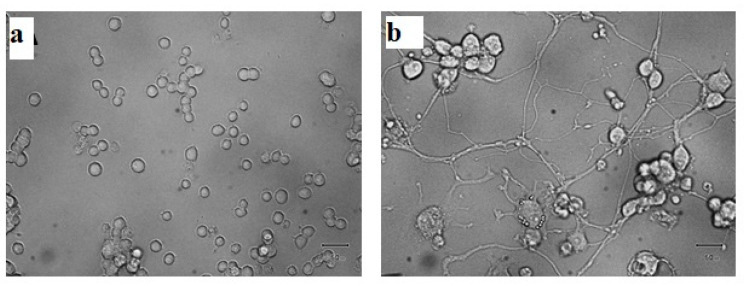
NGF-dependent neuronal phenotype differentiation of PC12 cells. Representative photomicrographs obtained by microscopy of PC12 cells untreated (**a**) or treated with 50 ng/mL NGF (**b**) for 72 h. Scale bar = 50 μm.

Neural differentiation of PC12 has been widely used in neuroscience both in neurobiological and neurotoxicological studies as a neuronal cell model [28]. Following treatment with nerve growth factor (NGF) PC12 cells exhibit a typical phenotype of neuronal cells, sending out neurites and acquiring several features typical of sympathetic neurons. To address this, PC12 cells were treated with NGF over the course of one week to allow for neuronal differentiation [29]. Figure 8 shows the effect of 50 ng/mL NGF on PC12 cells and as expected, NGF activated the cells differentiation, forming a complex neuronal network.

**Figure 9:**
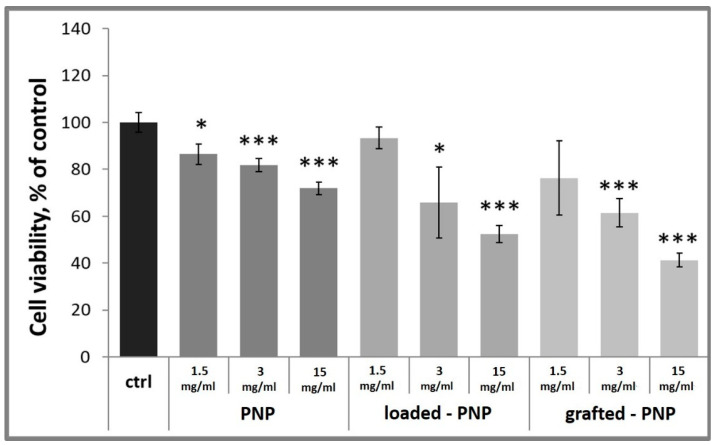
Dose-response experiment on d-PC12 cells. Cells were incubated for 24 h with PNPs. Results are presented as mean ± SD and normalized with respect to the control untreated cells. (* *p* < 0.05; *** *p* < 0.001 versus control untreated cells, one-way ANOVA).

To gain information on the biocompatibility of labelled of PNPs, loaded-PNPs and grafted-PNPs, d-PC12 cells were treated with them at different concentrations (15, 3, 1.5 mg/mL) for 24 h. Cell-viability data are reported in Figure 9. Figure 9 also shows that the unlabeled NPs’ addition to the culture medium significantly decreased the number of cells at all studied concentrations, 15, 3 and 1.5 mg/mL (86 ± 4%, 81 ± 3% and 72 ± 3% of untreated control). It is important to note, both loaded-PNPs and grafted-PNPs did not show any significant difference at concentration 1.5 mg/mL, compared with the untreated control 93 ± 5% and 76 ± 15%, respectively. At 3 and 15 mg/mL, both labelled PNPs induced a concentration-dependent decrease of PC12 cell viability, up to 66 ± 15%, 61 ± 6% for 3 mg/mL, and 52 ± 4%, 41 ± 3% for 1.5 mg/mL, respectively. We tested very high concentrations of NPs to simulate a potential accumulation of them in neuronal cells after more of one administration.

To understand if the toxicity of the studied PNPs depended on RhB’s presence, we examined RhB at the different concentration (2, 4, 20 µM). It is important to note, that RhB at all tested concentrations did not decrease cell viability after 24 h of treatment (data not shown).

It has been reported that PC12 is a cell line that has been widely used as an in vitro model for investigating the neuronal oxygen sensor mechanism [30].

**Figure 10:**
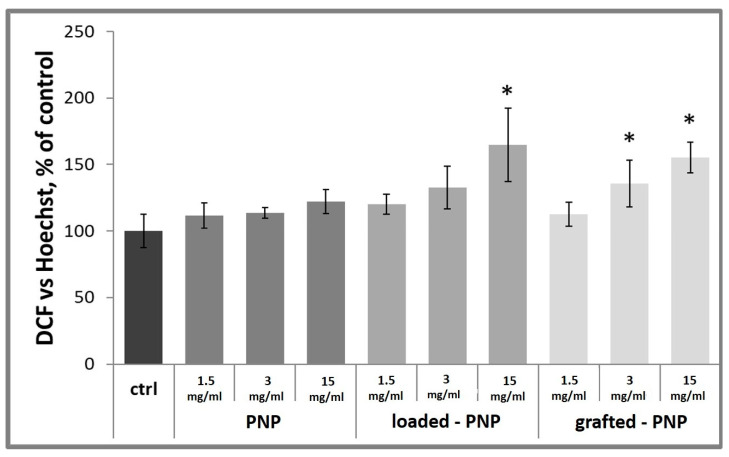
Detection of total ROS production in d-PC12 cells using DCF. Cells were treated 24 h with NPs and then analyzed by a Varioscan multimode microplate reader. Results are represented as the increase in DCF, normalized with live-cell fluorescent staining of DNA Hoechst33342, with respect to untreated control cells. Results are presented as mean ± SD. (* *p* < 0.05 versus PEG–PLGA NP-3, one-way ANOVA).

Here, intracellular ROS production was studied by using an oxidation-sensitive fluorescent probe dye (DCF), run to verify the damaging effects of NPs in the chosen neuron cell model. To quantify the level of intracellular ROS, such as H_2_O_2_, ·OH– and ONOO·–, we controlled DCF fluorescence, that, afterward, was normalized with Hoechst33342 nuclear stain to quantify the cell number for every well. Normal d-PC12 cells exhibited weak green fluorescence in the control group and the unloaded PNP group (data not shown). Results, shown in Figure 10, clearly demonstrated that loaded-PNPs and grafted-PNPs injured d-PC12 cells, depict enhanced green fluorescence, implying ROS accumulation in the injured cells. Especially, after the treatment with 3 and 1.5 mg/mL (polymer concentration in PNPs) of RhB-functionalized NPs, the DCF fluorescence intensity was increased after 24 h treatment up to 133 ± 16% and 165 ± 28% for loaded-PNPs and 136 ± 17% and 155 ± 11% for grafted-PNPs. It is important to note that incubation with RhB did not show a damaging effect for the cells (data not shown).

It has been shown that RhB, due to the positive charges in its structure, is able to bind to certain DNA sequences, potentially causing genotoxicity. DNA damage stimulates ROS production in cell cultures [31,32]. ROS accumulation in cells treated with loaded- and grafted-PNPs at the highest concentrations could be attributed to a more effective internalization of the dye when delivered into the NPs [33].

**Figure 11:**
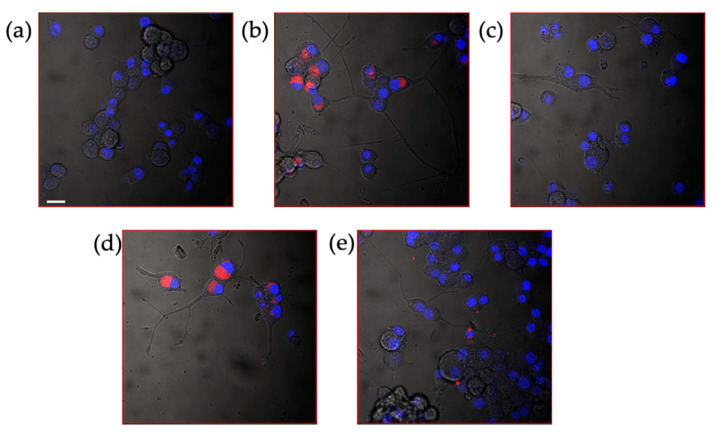
LSM-merged micrographs of bright field (in grey) and fluorescence (blue: Hoechst33342 nuclear staining, λex/em = 405/425–450 nm; red: Rhodamine B, λex/em = 543/550–600 nm) of d-PC12 cells untreated (negative control, (**a**) and after 2 h of treatment with: 4 µM RhB (positive control, (**b**), 3 mg/mL bare PNP (positive control, (**c**), 3 mg/mL RhB loaded-PNP (**d**), 3 mg/mL RhB grafted-PNP (**e**). Scale bar 20 µm.

Laser scanning confocal microscopy (LSM) analyses were carried out to image the actual internalization of loaded- and grafted-PNPs by the d-PC12 cells (Figure 11). Untreated cells (Figure 11a) and cells treated for 2 h with RhB (Figure 11b) or bare PNPs (Figure 11c) were added into the study as negative and positive controls, respectively.

A similar diffuse red emission was clearly visible in the cell cytoplasm for both cells treated with the fluorescent dye alone and those treated with RhB-loaded PNPs (Figure 11d). On the other hand, the cells treated with RhB-grafted PNPs (Figure 11e) exhibited a specked, but still evident, red fluorescence, which was not observed in the cells untreated nor in those treated with bare PNPs.

**Figure 12:**
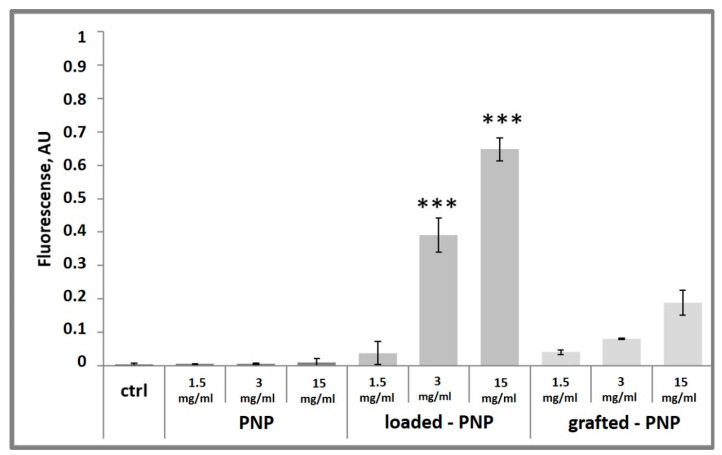
Detection of intracellular RhdB emission in d-PC12 cells. Cells were treated 24 h with NPs or RhB (data not shown) and then analyzed by a Varioscan multimode microplate reader. Results are represented as the increase in fluorescence, normalized with live-cell fluorescent staining of DNA Hoechst33342, with respect to untreated control cells. Results are presented as mean ± SD. (*** *p* < 0.001 versus grafted-PNP, one-way ANOVA).

The internalization of the labelled PNPs with the fluorescent dye RhB was quantified in d-PC12 cells exposed to 15, 3 and 1.5 mg/mL dilutions of each nanoparticle sample (Figure 12). It is known that RhB crosses biological membranes and can be used in microfluidic and microscopic applications [34]. In accordance with our results (Figure 4 and Figure 5) and literature data, we analyzed d-PC12-treated cells for RhB intracellular fluorescence with an absorption peak centered at 554 nm, and an emission peak at 576 nm [35]. As expected, control- and PNP-treated cells did not show any emission in red spectra. Figure 11 demonstrated that after the 24 h of incubation with both loaded- and grafted-PNPs increased the intracellular levels of fluorescence at 576 nm. Important to underline that emission was significantly higher for loaded-PNPs than for grafted-PNPs at all tested concentrations, because each type of formulation incorporated different amounts of dye. Our results suggested that both nanosystems are efficient for in vitro/in vivo investigations.

## 4. Conclusions

In this work, we have produced Rhodamine (RhB)-labelled NPs for in vitro/in vivo fluorescence imaging studies, a potentially useful carrier to investigate the nose-to-brain delivery administration route of drugs. The studied NPs were obtained by the nanoprecipitation method and by following two different strategies to entrap RhB into the PEG–PLGA nanoparticles (PNPs), i.e., the physical entrapment of free dye into the particles (loaded-PNPs) or the use of a graft copolymer, where RhB was covalently linked on the polymeric backbone (grafted-PNPs). The latter grafted polymer was obtained by covalent binding of the RhB on a polyaspartamide/polylactide graft copolymer backbone. The involvement of RhB in a covalent linkage was confirmed by UV-vis characterization studies on the polymer matrix, which showed a slightly shifted UV-vis peak compared to the loaded-PNPs matrix [20]. The obtained systems were characterized by PCS analysis showed suitable size for nose-to-brain delivery (<200 nm), homogeneous particle populations with PDI < 0.3, a negative surface charge (−23.4 mV) and high PEGylation density with brush-like conformation in all investigated systems. The most appropriate purification process was centrifugation, which allowed removal of approximately 40% of the unencapsulated dye and allowed us to obtain a zero-dye release profile up to 72 h for loaded-PNPs. On the other hand, the labeling method of the grafted-PNPs, i.e., by the entrapment of RhB stably linked on the PHEA–*g*–RhB–*g*–PLA backbone, ensured the stability of the fluorescent dye inside the matrix, avoiding any diffusion process. Cell-viability tests on OEC cells showed the absence of cytotoxicity at all tested concentrations. Dose-dependent toxicity was observed in vitro studies on d-PC12 cells and was higher for grafted-PNPs than for loaded-PNPs. Although cytotoxicity studies revealed that loaded-PNPs showed lower cytotoxicity compared with grafted-PNPs, the level of ROS production was rather comparable between both investigated systems. Moreover, by in vitro studies we have demonstrated that both PNPs are internalized from cells as the endocellular fluorescence increases, as a function of the concentration of PNPs in the incubation medium. In conclusion, both RhB labelled nanocarriers described in this paper proved to be useful for potential in vitro/in vivo imaging after intranasal administration.

## Figures and Tables

**Table 1:** Chemicophysical properties and yield of purification of loaded-PNPs purified through centrifugation and dialysis procedures.

Type and Purification Processes Steps		Z-ave (nm) ± S.D. ^b^	PDI ± S.D. ^b^	ZP ± S.D. ^b^	%EE ^c^	%Yield of Purification Respect to the Unloaded RhB
Before purification process		344.5 ± 9.9	0.098 ± 0.035	−1.4 ± 0.1	80.190 ± 8.234	
After purification process	Dialysis	143.6 ± 2.2	0.135 ± 0.018	−20.2 ± 0.9		3.857± 1.241
	A.C. ^a^ 1 step	145.9 ± 0.4	0.117 ± 0.005	−23.4 ± 0.9		34.148 ± 1.293 ^d^
	A.C. ^a^ final step	190.2 ± 4.1	0.198 ± 0.024	−27.7 ± 0.3		40.754 ± 2.113 ^d^

^a^ A.C.—after centrifugation; ^b^ S.D.—standard deviation; ^c^ E.E.—encapsulation efficiency; ^d^ total sum of the three centrifuge steps.

**Table 2:** Amount of PEG moieties on the NP surface, surface PEG density [Γ]. Number of PEG per 100 nm^2^ and ratios of PEG density to full surface coverage [Γ/Γ*] of PNP, loaded-PNPs and grafted-PNPs. Sample PEG amount on NP surface (mmol/100 mg) [Γ] (PEG chains/100 nm^2^).

Sample	PEG Amount on NP Surface (mmol/100 mg)	[Γ] (PEG Chains/100 nm^2^)	Γ/Γ*
**PNP**	2.79 × 10^−4^	13.7	3.1
**Loaded-PNP**	3.99 × 10^−4^	8.4	1.9
**Grafted-PNP**	4.86 × 10^−^^4^	17.4	4.0

## Data Availability

The data presented in this study are available on request from the corresponding author through email: teresa.musumeci@unict.it.

## Supplementary Materials

The following are available online at https://www.mdpi.com/article/10.3390/pharmaceutics13091508/s1, Figure S1: UV-vis calibration curve for RhB in Milli-Q water determined at the wavelength of the maximum of absorbance (λ = 552 nm). Figure S2: DSC thermograms of PLGA–PEG (red curve), PHEA–*g*–RhB–*g*–PLA (black curve), RhB (blue curve) as raw materials, unlabeled PNPs (green curve), loaded-PNPs (yellow curve) and grafted-PNPs (brown curve).
