# Supplementary material for: mPEG-PLGA Nanoparticles Labelled with Loaded or Conjugated Rhodamine-B for Potential Nose-to-Brain Delivery
Source: Pharmaceutics. 2021 Sep 18;13(9):1508. doi: 10.3390/pharmaceutics13091508 (PMC8471208; doi:10.3390/pharmaceutics13091508)
Supplement: Supplementary file 1 [file pharmaceutics-13-01508-s001.zip › pharmaceutics-1351906-supplementary.pdf]

pharmaceutics

mPEG-PLGA Nanoparticles Labelled with Loaded or Conjugated Rhodamine-B for Potential Nose-to-Brain Delivery

Emanuela Fabiola Craparo, Teresa Musumeci, Angela Bonaccorso, Rosalia Pellitteri, Alessia Romeo, Irina Naletova, Lorena Maria Cucci, Gennara Cavallaro and Cristina Satriano

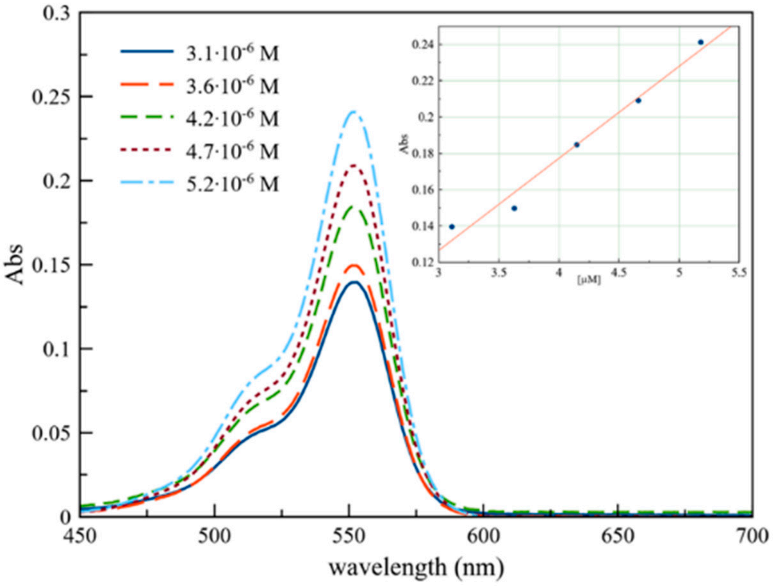

Figure S1. UV-vis calibration curve for RhB in Milli-Q water determined at the wavelength of the maximum of absorbance (λ = 552 nm).

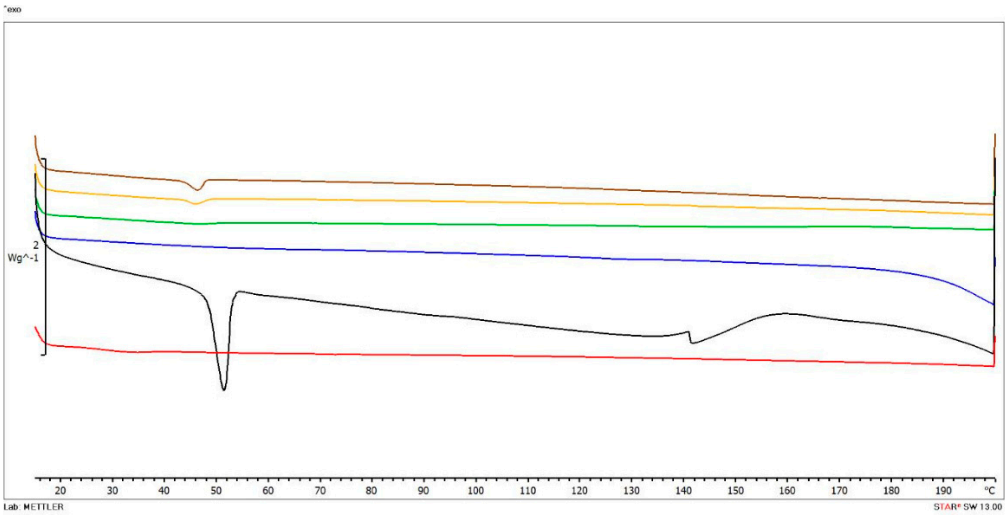

Figure S2. DSC thermograms of PLGA–PEG (red curve), PHEA–*g*–RhB–*g*–PLA (black curve), RhB (blue curve) as raw materials, unlabeled PNPs (green curve), loaded-PNPs (yellow curve) and grafted-PNPs (brown curve).
